# Supplementary material for: Response of treatment-naive brain metastases to stereotactic radiosurgery
Source: Nat Commun. 2024 May 2;15:3728. doi: 10.1038/s41467-024-47998-8 (PMC11066027; doi:10.1038/s41467-024-47998-8)
Supplement: Supplementary file 1 — Supplementary Information [file 41467_2024_47998_MOESM1_ESM.pdf]

Supplemental Figure S1. Impact of tumor histology on local control

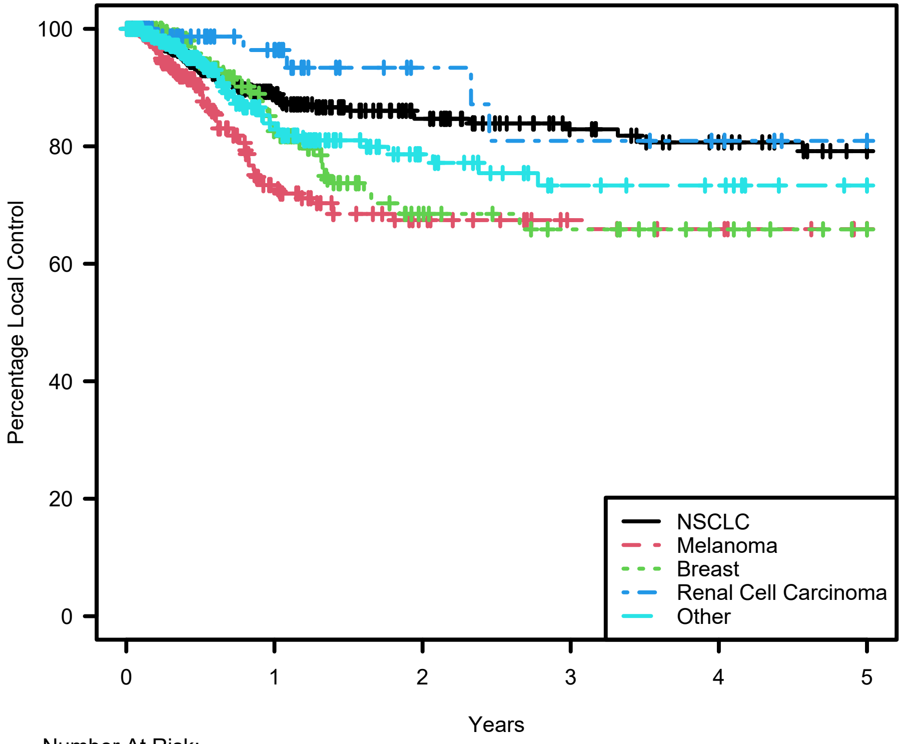

Number At Risk:

NSCLC	628	212	125	83	63	46
Melanoma	355	104	58	46	42	33
Breast	221	97	35	23	16	10
Renal	103	41	18	14	11	8
Other	426	122	55	32	27	18

Supplemental Figure S1. Impact of tumor histology on local control. Kaplan-Meier curves showing percent local control of brain metastasis lesions over time after Stereotactic Radiosurgery (SRS) stratified by histology. *x-axis is censored at 5 years post-SRS.* Abbreviations: NSCLC- Non-small cell lung cancer. Source data provided as a Source data file.

Supplemental Figure S2. Impact of Age on local control

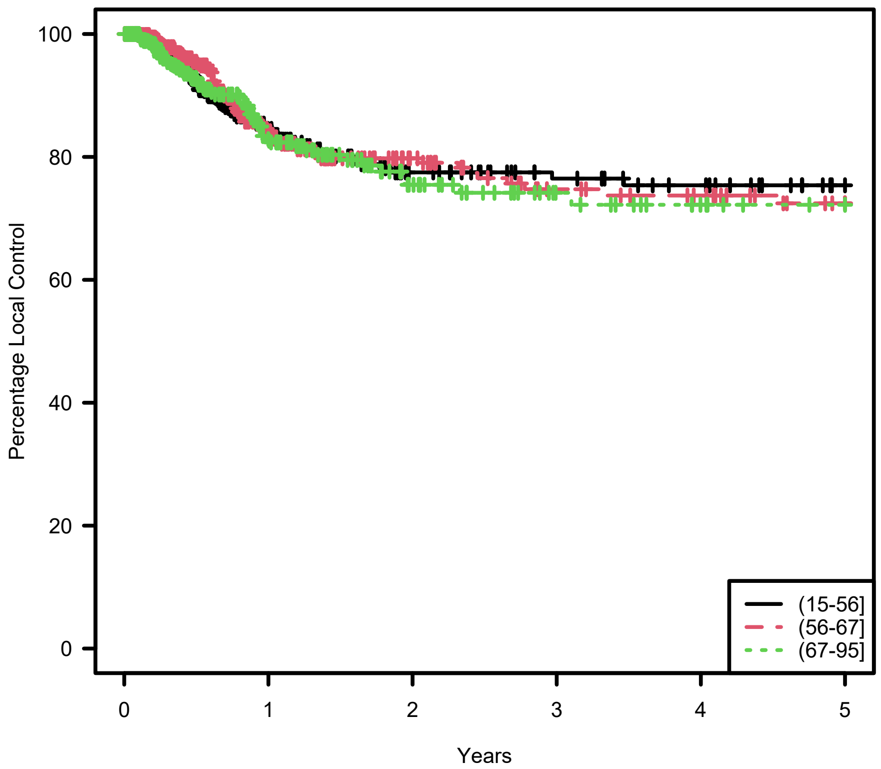

Number At Risk:

(15-56]	579	200	106	77	66	51
(56-67]	603	214	112	77	69	51
(67-95]	550	159	69	39	21	13

Supplemental Figure S2. Impact of Age on local control. Kaplan-Meier curves showing percent local control of brain metastasis lesions over time after Stereotactic Radiosurgery (SRS) stratified by age. *x-axis is censored at 5 years post-SRS as only 5 lesions under surveillance failed SRS after 5 years.* Source data provided as a Source data file.

Supplemental Figure S3. Impact of Sex on local Control

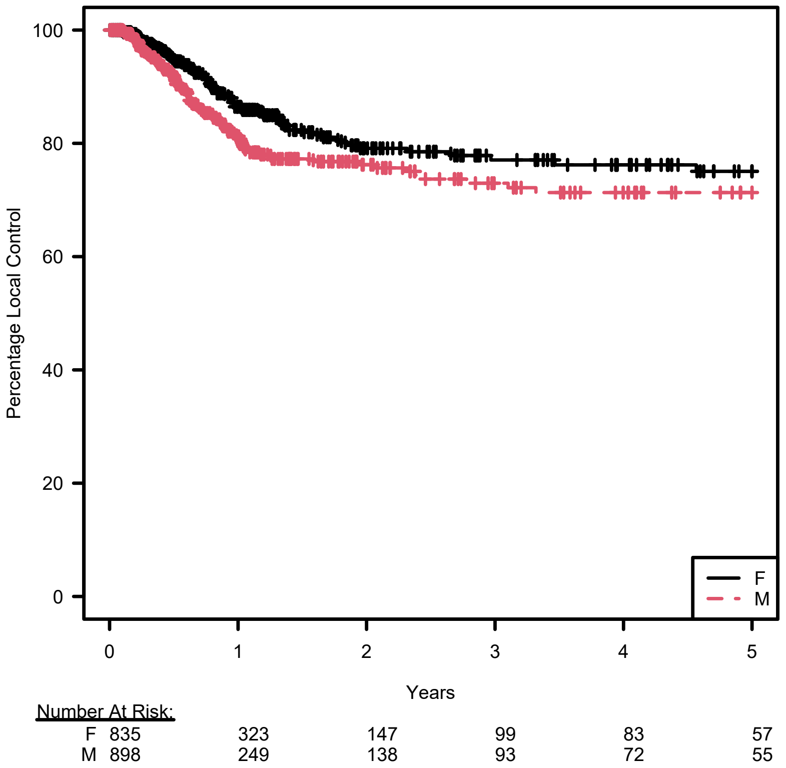

Supplemental Figure S3. Impact of Sex on local Control. Kaplan-Meier curves showing percent local control of brain metastasis lesions over time after Stereotactic Radiosurgery (SRS) stratified by sex. *x-axis is censored at 5 years post-SRS as only 5 lesions under surveillance failed SRS after 5 years. Abbreviations: F-Female, M-Male.* Source data provided as a Source data file.

Supplemental Figure S4. Low cerebral blood volume in case of radiation necrosis

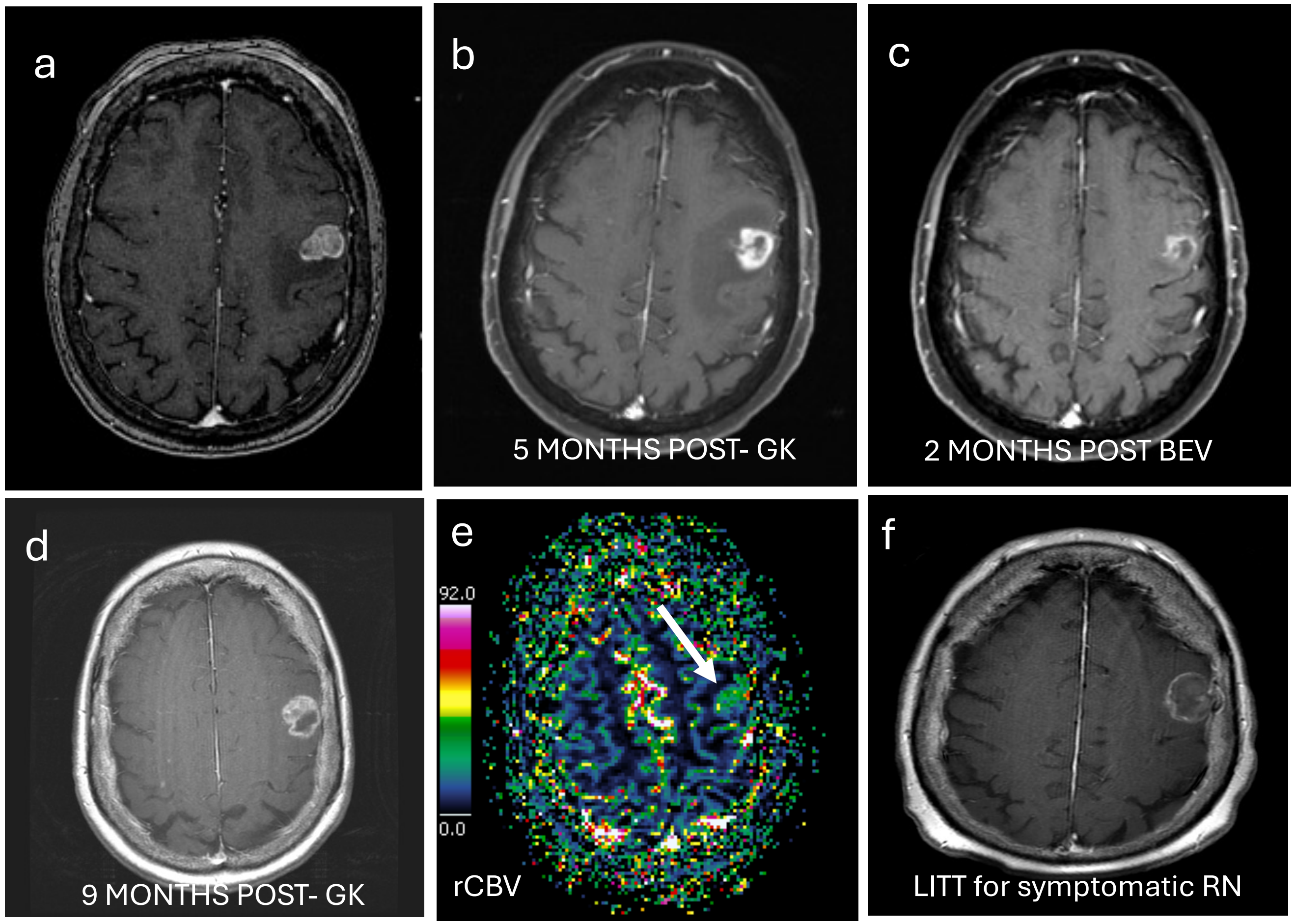

Supplemental Figure S4. Low cerebral blood volume in case of radiation necrosis. Magnetic Resonance images of a patient presenting with aphasia who received GK Stereotactic Radiosurgery (SRS) to a left frontal lesion (a). Following cessation of steroids, there was increased perilesional edema and enhancement (b). Bevacizumab (BEV) was initiated with response noted (c). Following cessation of Bevacizumab, there was an increase in enhancement (d), with a low relative cerebral blood volume (rCBV; white arrow in e) consistent with recurrence of pseudoprogression following discontinuation of Bevacizumab. Given that patient was symptomatic with worsening aphasia, Laser Interstitial Thermal Therapy (LITT) was administered to the lesion with radiographic and clinical improvement noted (f; 3 months post-LITT). Abbreviations: GK, Gamma Knife; BEV, Bevacizumab; CBV, Cerebral blood volume; LITT, laser Interstitial Thermal Therapy.
