# Supplementary material for: Response of treatment-naive brain metastases to stereotactic radiosurgery
Source: Nat Commun. 2024 May 2;15:3728. doi: 10.1038/s41467-024-47998-8 (PMC11066027; doi:10.1038/s41467-024-47998-8)
Supplement: Supplementary file 3 — Reporting Summary [file 41467_2024_47998_MOESM3_ESM.pdf]

nature portfolio

Corresponding author(s): Chibawanye Ene

Last updated by author(s): March 17, 2024

Reporting Summary

Nature Portfolio wishes to improve the reproducibility of the work that we publish. This form provides structure for consistency and transparency in reporting. For further information on Nature Portfolio policies, see our Editorial Policies and the Editorial Policy Checklist.

Please do not complete any field with "not applicable" or n/a. Refer to the help text for what text to use if an item is not relevant to your study.
For final submission: please carefully check your responses for accuracy; you will not be able to make changes later.

Statistics

For all statistical analyses, confirm that the following items are present in the figure legend, table legend, main text, or Methods section.

n/a	Confirmed	
☐	☑	The exact sample size (*n*) for each experimental group/condition, given as a discrete number and unit of measurement
☐	☑	A statement on whether measurements were taken from distinct samples or whether the same sample was measured repeatedly
☐	☑	The statistical test(s) used AND whether they are one- or two-sided<br>*Only common tests should be described solely by name; describe more complex techniques in the Methods section.*
☐	☑	A description of all covariates tested
☐	☑	A description of any assumptions or corrections, such as tests of normality and adjustment for multiple comparisons
☐	☑	A full description of the statistical parameters including central tendency (e.g. means) or other basic estimates (e.g. regression coefficient) AND variation (e.g. standard deviation) or associated estimates of uncertainty (e.g. confidence intervals)
☐	☑	For null hypothesis testing, the test statistic (e.g. *F*, *t*, *r*) with confidence intervals, effect sizes, degrees of freedom and *P* value noted<br>*Give P values as exact values whenever suitable.*
☒	☐	For Bayesian analysis, information on the choice of priors and Markov chain Monte Carlo settings
☒	☐	For hierarchical and complex designs, identification of the appropriate level for tests and full reporting of outcomes
☒	☐	Estimates of effect sizes (e.g. Cohen's *d*, Pearson's *r*), indicating how they were calculated

Our web collection on statistics for biologists contains articles on many of the points above.

Software and code

Policy information about availability of computer code

Data collection	Data was collected by reviewing patient charts manually and documenting independent radiologist official report of changes to treated brain metastasis
Data analysis	Statistical analyses were performed using R statistical software.8 All statistical tests utilized two-sided alpha = 0.05 for a 95% level of statistical confidence.<br>Survival modeling was performed using the “survival” package.9,10 Assessment of differences among discrete variable levels in the accelerated failure time model were estimated using the “emmeans” package which includes adjusted means weighted proportionally to covariate marginal frequencies

For manuscripts utilizing custom algorithms or software that are central to the research but not yet described in published literature, software must be made available to editors and reviewers. We strongly encourage code deposition in a community repository (e.g. GitHub). See the Nature Portfolio guidelines for submitting code & software for further information.

Data

Policy information about availability of data

All manuscripts must include a data availability statement. This statement should provide the following information, where applicable:

- Accession codes, unique identifiers, or web links for publicly available datasets
- A description of any restrictions on data availability
- For clinical datasets or third party data, please ensure that the statement adheres to our policy

The data that support the findings of this study are not openly available given IRB restrictions on human clinical data. Anonymized data are available from the corresponding author upon reasonable request and IRB approval. Following IRB approval, the de-identified data will be made available within 2 weeks.

Research involving human participants, their data, or biological material

Policy information about studies with human participants or human data. See also policy information about sex, gender (identity/presentation), and sexual orientation and race, ethnicity and racism.

Reporting on sex and gender	Sex is now reported. 507 females and 588 male patients were included and reported in Table 3 and Supplemental figure S3. Sex is defined according to the reproductive organs and functions that derive from chromosomal complement
Reporting on race, ethnicity, or other socially relevant groupings	Information on race and ethnicity was collected as part of retrospective analysis
Population characteristics	Population characteristics are listed on Table 3
Recruitment	All analysis in this paper was collected retrospectively, therefore no recruitment occured.
Ethics oversight	The institutional Review Board (IRB) at the University of Texas M D Anderson Cancer Center (UTMDACC) approved this retrospective study which included chart review of 3000 patients with metastatic brain lesions treated with the frame-based LINAC and Gamma Knife (GK) SRS over 25 years at the UTMDACC from 1993 to 2018

Note that full information on the approval of the study protocol must also be provided in the manuscript.

Field-specific reporting

Please select the one below that is the best fit for your research. If you are not sure, read the appropriate sections before making your selection.

☑ Life sciences ☐ Behavioural & social sciences ☐ Ecological, evolutionary & environmental sciences

For a reference copy of the document with all sections, see nature.com/documents/nr-reporting-summary-flat.pdf

Life sciences study design

All studies must disclose on these points even when the disclosure is negative.

Sample size	No sample size was calculated. We report on all the cases within the specific time frame that met critiera for inculsion.
Data exclusions	Patients or lesions with prior surgery, radiation, systemic treatment, size >3cm, 4 or more tumors, no post-op Imaging, lost to follow-up.
Replication	Since this is a retrospective analysis from a single institution, we have provided all parameters used to aqcuire the tumor response in figure 1 to ensure reproducibility
Randomization	Randomization is not applicable because this retrospective study was conceived after treatment and outcomes occured. Also there was only 1 type of treatment given and it was the standard of care for brain mets.
Blinding	Blinding is not applicable because the treatment and results reported were already documented by independent radiologist prior to conceiving this study.

Behavioural & social sciences study design

All studies must disclose on these points even when the disclosure is negative.

Study description	Not a behavioural or social science study
Research sample	Not a behavioural or social science study
Sampling strategy	Not a behavioural or social science study
Data collection	Not a behavioural or social science study
Timing	Not a behavioural or social science study
Data exclusions	Not a behavioural or social science study
Non-participation	Not a behavioural or social science study
Randomization	Not a behavioural or social science study

Ecological, evolutionary & environmental sciences study design

All studies must disclose on these points even when the disclosure is negative.

Study description	Not an ecological, evolutionary and environmental science study
Research sample	Not an ecological, evolutionary and environmental science study
Sampling strategy	Not an ecological, evolutionary and environmental science study
Data collection	Not an ecological, evolutionary and environmental science study
Timing and spatial scale	Not an ecological, evolutionary and environmental science study
Data exclusions	Not an ecological, evolutionary and environmental science study
Reproducibility	Not an ecological, evolutionary and environmental science study
Randomization	Not an ecological, evolutionary and environmental science study
Blinding	Not an ecological, evolutionary and environmental science study

Did the study involve field work? ☐ Yes ☒ No

Field work, collection and transport

Field conditions	Not field work
Location	Not field work
Access & import/export	Not field work
Disturbance	Not field work

Reporting for specific materials, systems and methods

We require information from authors about some types of materials, experimental systems and methods used in many studies. Here, indicate whether each material, system or method listed is relevant to your study. If you are not sure if a list item applies to your research, read the appropriate section before selecting a response.

Materials & experimental systems

n/a	Involved in the study	
☒	☐	Antibodies
☒	☐	Eukaryotic cell lines
☒	☐	Palaeontology and archaeology
☒	☐	Animals and other organisms
☐	☑	Clinical data
☒	☐	Dual use research of concern
☒	☐	Plants

Methods

n/a	Involved in the study	
☒	☐	ChIP-seq
☒	☐	Flow cytometry
☐	☑	MRI-based neuroimaging

Antibodies

Antibodies used	No antibodies were used
Validation	No antibodies were used

Eukaryotic cell lines

Policy information about cell lines and Sex and Gender in Research

Cell line source(s)	No cell lines were used
Authentication	No cell lines were used
Mycoplasma contamination	No cell lines were used
Commonly misidentified lines (See ICLAC register)	No cell lines were used

Palaeontology and Archaeology

Specimen provenance	Not a palaeontology or archaeology study
Specimen deposition	Not a palaeontology or archaeology study
Dating methods	Not a palaeontology or archaeology study

☐ Tick this box to confirm that the raw and calibrated dates are available in the paper or in Supplementary Information.

Ethics oversight	Not a palaeontology or archaeology study

Note that full information on the approval of the study protocol must also be provided in the manuscript.

Animals and other research organisms

Policy information about studies involving animals; ARRIVE guidelines recommended for reporting animal research, and Sex and Gender in Research

Laboratory animals	No animals were used in this study
Wild animals	No animals were used in this study
Reporting on sex	No animals were used in this study
Field-collected samples	No animals were used in this study
Ethics oversight	No animals were used in this study

Note that full information on the approval of the study protocol must also be provided in the manuscript.

Clinical data

Policy information about clinical studies

All manuscripts should comply with the ICMJE guidelines for publication of clinical research and a completed CONSORT checklist must be included with all submissions.

Clinical trial registration	Not a clinical trial. This is a retrospective study.
Study protocol	Impact of Tumor Size on Patient Outcomes Following Stereotactic Radiosurgery for Brain Metastasis (2021-0698)
Data collection	Chart review to collect baseline characteristics, demographics, radiation treatment parameters, sequence of radiation therapy, imaging data
Outcomes	Not a human clinical trial. This is a retrospective study evaluating scans and survival after SRS over a specific period of time

Dual use research of concern

Policy information about dual use research of concern

Hazards

Could the accidental, deliberate or reckless misuse of agents or technologies generated in the work, or the application of information presented in the manuscript, pose a threat to:

No	Yes	
☒	☐	Public health
☒	☐	National security
☒	☐	Crops and/or livestock
☒	☐	Ecosystems
☒	☐	Any other significant area

Experiments of concern

Does the work involve any of these experiments of concern:

No	Yes	
☒	☐	Demonstrate how to render a vaccine ineffective
☒	☐	Confer resistance to therapeutically useful antibiotics or antiviral agents
☒	☐	Enhance the virulence of a pathogen or render a nonpathogen virulent
☒	☐	Increase transmissibility of a pathogen
☒	☐	Alter the host range of a pathogen
☒	☐	Enable evasion of diagnostic/detection modalities
☒	☐	Enable the weaponization of a biological agent or toxin
☒	☐	Any other potentially harmful combination of experiments and agents

Plants

Seed stocks	No plants were used
Novel plant genotypes	No plants were used
Authentication	No plants were used

ChIP-seq

Data deposition

☐ Confirm that both raw and final processed data have been deposited in a public database such as GEO.

☐ Confirm that you have deposited or provided access to graph files (e.g. BED files) for the called peaks.

Data access links<br>*May remain private before publication.*	Chip-Seq was not performed
Files in database submission	Chip-Seq was not performed
Genome browser session<br>(e.g. UCSC)	Chip-Seq was not performed

Methodology

Replicates	Chip-Seq was not performed
Sequencing depth	Chip-Seq was not performed
Antibodies	Chip-Seq was not performed
Peak calling parameters	Chip-Seq was not performed
Data quality	Chip-Seq was not performed
Software	Chip-Seq was not performed

Flow Cytometry

Plots

Confirm that:

☐ The axis labels state the marker and fluorochrome used (e.g. CD4-FITC).

☐ The axis scales are clearly visible. Include numbers along axes only for bottom left plot of group (a 'group' is an analysis of identical markers).

☐ All plots are contour plots with outliers or pseudocolor plots.

☐ A numerical value for number of cells or percentage (with statistics) is provided.

Methodology

Sample preparation	Flow cytometry was not performed
Instrument	Flow cytometry was not performed
Software	Flow cytometry was not performed
Cell population abundance	Flow cytometry was not performed
Gating strategy	Flow cytometry was not performed

☐ Tick this box to confirm that a figure exemplifying the gating strategy is provided in the Supplementary Information.

Magnetic resonance imaging

Experimental design

Design type	Retrospective study, MRI of the brain from patient charts was used
Design specifications	No specific experimental setup was used
Behavioral performance measures	No behavioural measures were included

Imaging type(s)	T1 weighted MRI with contrast obtained before and after SRS treatment were used for measurements
Field strength	We acquire 1.5T and 3T scans. Most of the 1.5T scans are acquired on MR22 & MR23 – Siemens Aera.<br>Most of the 3T scans are acquired on the PET/MR -- a GE SIGNA.
Sequence & imaging parameters	T1 weighted Fast 3D gradient echo, 1 mm axial images, FOV 256 mm, gadolinium contrast
Area of acquisition	Whole brain to C2 vertebra

Diffusion MRI ☑ Used ☐ Not used

Preprocessing

Preprocessing software	
Normalization	
Normalization template	
Noise and artifact removal	3D distortion correction
Volume censoring	None

Statistical modeling & inference

Model type and settings	Time to treatment failue (TTF) was modeled by accelerated failure time models with log-logistic distribution selected per Akaike Information Criteria among Weibull, hat exponential, Gaussian, logistic, log-normal, and log-logistic distributions, and verified by residual plot overlaid on the distribution as well as deviance residual plots for covariates, and clustering on patients to control for repeated events. Model-adjusted differences among the levels of discrete variables in TTF were assessed by Tukey-adjustedcontrasts. Survival modeling was performed using the "survival" package in R software
Effect(s) tested	None

Specify type of analysis: ☐ Whole brain ☑ ROI-based ☐ Both

Statistic type for inference

(See Eklund et al. 2016)

None

Correction

None

Models & analysis

n/a	Involved in the study
☒	☐ Functional and/or effective connectivity
☐	☑ Graph analysis
☐	☑ Multivariate modeling or predictive analysis

Functional and/or effective connectivity

Not performed

Graph analysis

Time to treatment failure (TTF) was summarized by Kaplan-Meier methods for discrete variables

Multivariate modeling and predictive analysis

Multivariate analysis methods are described with references in methods. Assessment of differences among discrete variable levels in the accelerated failure time model were estimated using the "emmeans" package. Satistical analysis were performed using R statistical software

This checklist template is licensed under a Creative Commons Attribution 4.0 International License, which permits use, sharing, adaptation, distribution and reproduction in any medium or format, as long as you give appropriate credit to the original author(s) and the source, provide a link to the Creative Commons license, and indicate if changes were made. The images or other third party material in this article are included in the article's Creative Commons license, unless indicated otherwise in a credit line to the material. If material is not included in the article's Creative Commons license and your intended use is not permitted by statutory regulation or exceeds the permitted use, you will need to obtain permission directly from the copyright holder. To view a copy of this license, visit http://creativecommons.org/licenses/by/4.0/
